# Case Managers' Perceptions About Synchronous Telerehabilitation versus Clinic-based Physical Therapy Services for People with Spinal Cord Injury

**DOI:** 10.5195/ijt.2021.6392

**Published:** 2021-12-16

**Authors:** Steve Kerschke, Karen Hux

**Affiliations:** Kintinu Telerehab, Quality Living, Inc., Omaha, Nebraska, USA

**Keywords:** Physical therapy, Service delivery models, Spinal cord injury, Synchronous telerehabilitation, Telehealth

## Abstract

People with spinal cord injury (SCI) require extensive rehabilitation to maximize independence and quality of life. Much of this treatment occurs on an outpatient basis through telerehabilitation or clinic-based services. Synchronous telerehabilitation has become increasingly common in recent years, but many professionals remain reluctant to suggest it when clinic-based services are available. This survey study explored case managers' perceptions regarding advantages and disadvantages of synchronous telerehabilitation versus clinic-based physical therapy services for people with SCI. Respondents were 89 case managers responsible for service provision coordination. Results showed a significant preference for clinic-based rather than telerehabilitation physical therapy services. Relative experience with the two service delivery models significantly affected perceptions. Only facilitating travel convenience differed significantly as a reason for recommending one service delivery method over the other. The incongruity between perceptions about synchronous telerehabilitation and existing literature about its cost, convenience, and efficacy suggests a need for additional education.

Synchronous telerehabilitation uses telecommunication technologies to allow rehabilitation specialists to assess and treat people at physically distant locations by engaging in real-time, two-way audiovisual interactions (American Telemedicine Association, 2020). Although originally conceptualized to make services available to people living far away from brick-and-mortar facilities, telerehabilitation has expanded in scope and implementation in recent years, particularly with the necessity of limiting close personal interactions during the worldwide spread of COVID-19 (Lee, 2020; Stein et al., 2020; Turolla et al., 2020). However, persisting concerns and misperceptions may make some professionals reluctant to recommend telerehabilitation as a service delivery model for certain services—such as physical therapy—for people needing continued treatment following inpatient discharge. Although clients indicate generalized acceptance of and satisfaction with telerehabilitation services (Harkey et al., 2020; Miller et al., 2020; Yuen et al., 2015), professionals remain more skeptical (Sineus & Capellan, 2019). Reluctance to endorse fully telerehabilitation services is likely to affect the frequency with which the option is offered to persons needing outpatient treatment.

## TELEREHABILITATION ADVANTAGES AND DISADVANTAGES

Widely accepted advantages exist for the use of telerehabilitation rather than the more traditional, clinic-based service delivery model. These advantages include the possibility for consultation with top professional specialists regardless of geographic proximity (Irgens et al., 2018; Niknamian, 2019; Schmeler et al., 2008), the elimination of travel time and expense for clients as well as reduced reliance on other people or handicapped-accessible transportation (Kairy et al., 2013; Levy et al., 2015; Nikmanian, 2019; Schmeler et al., 2009; Yuen et al., 2015), and the receipt of therapy services in the comfort and privacy of home (Kairy et al., 2013; Theodoros & Russell, 2008).

Disadvantages are also possible, however. For example, concerns persist about the adequacy of treatment programs planned without at least some in-person contact (Kairy et al., 2013; Theodoros & Russell, 2008). A second concern is that clients may struggle to operate telecommunication technologies and worry about the potential for equipment or connectivity problems interfering with sessions (Tsai et al., 2019). Third, some clients may miss the community support afforded by attending sessions with other people with similar conditions (Cranen et al., 2011; Turolla et al., 2020). Weighing the advantages and disadvantages for a particular client is critical to making appropriate service delivery recommendations.

Physical therapy has additional factors that may contribute to concerns about recommending its delivery via telerehabilitation rather than a clinic-based model. One such factor is the issue of overall treatment efficacy. To date, few researchers have performed well-designed investigations about whether clients achieve comparable benefit from remote and clinic-based physical therapy services. Much of the research that does exist implements a hybrid model of combined home-and clinic-based sessions rather than solely remote delivery (Levy et al., 2015; Neo et al., 2019). The rationale for this is that combined service delivery boosts confidence about diagnostic accuracy and progress assessment by providing periodic opportunities for physical contact and manipulation of a client's limbs and joints. However, regardless of implementation of combined or solely remote delivery, findings from studies comparing telerehabilitation and clinic-based physical therapy service delivery consistently suggest comparable results (Cramer et al., 2019; Levy et al., 2015; Neo et al., 2019; Turolla et al., 2020). Still, the perception that telerehabilitation services are inferior to clinic-based services may persist in the minds of some people responsible for making recommendations about service options.

Another concern about delivering physical therapy services via telerehabilitation relates to client safety because of the possibility of a person falling or sustaining injury while performing activities without a trained professional present. Research about this risk is scarce. However, Cramer et al. (2019) reported the frequency of adverse events relating to treatment was roughly comparable among adults with stroke receiving telerehabilitation (i.e., 6 of 59 people) and clinic-based services (i.e., 5 of 62 people); however, because the physical treatment targeted in this study was restoration of upper limb movement, falling was not likely for any study participant. Systematic investigations to determine whether risks exceed those associated with clinic-based services for various patient groups and types of physical challenges may provide further reassurance about the safety of telerehabilitation as a delivery model for physical therapy.

Other issues relate to (a) the adequacy of treatment personalization (Dunphy & Gardner, 2020), (b) the lack of specialty equipment available in home environments (Dunphy & Gardner, 2020; Turolla et al., 2020), and (c) reduced quality of client-therapist relationships (Anderson et al., 2017; Turolla et al., 2020). Additionally, some physical therapists lack familiarity using telerehabilitation technology (Sineus & Capellan, 2019; Theodoros & Russell, 2008) and, hence, are reluctant to adopt the service delivery model. Finally, the level of client independence and self-motivation needed for diligence and compliance with treatment protocols may be a concern for some clients and professionals (Dunphy & Gardner, 2020; Matamala-Gomez et al., 2020). In combination, issues such as these can contribute to skepticism among healthcare payors about recommending physical therapy services via telerehabilitation despite acknowledgement of inherent advantages.

## PHYSICAL THERAPY OUTPATIENT SERVICES FOR PEOPLE WITH SCI

People with spinal cord injury (SCI) have the potential to benefit substantially from physical therapy services. As a group, people with SCI often need extensive rehabilitation to maximize their mobility, independence, and quality of life. This treatment typically extends beyond inpatient rehabilitation to include some form of outpatient service (Nas et al., 2015; Whiteneck et al., 2011). Support for the importance of extensive intervention comes from research documenting continued gains in independence and quality of life following inpatient discharge when people with SCI receive outpatient rehabilitation (Derakhshanrad et al., 2015).

Literature about synchronous telerehabilitation services suggests that the benefit people experience from outpatient treatment is not dependent on the method of delivery (Cramer et al., 2019; Levy et al., 2015; Neo et al., 2019; Turolla et al., 2020). However, aside from the period during which limitations to in-person contact have occurred because of the COVID-19 pandemic, the most common method of outpatient service delivery for people with SCI has been clinic-based. Whether the provision of physical therapy through telerehabilitation for people with SCI remains a common option after resolution of the pandemic will depend, at least in part, on the willingness of professionals to endorse and recommend the service delivery option. This is likely to happen only if professionals perceive telerehabilitation and clinic-based service delivery models as comparable options regarding cost, convenience, and efficacy—as has been documented in existing research.

## STUDY PURPOSE

Workers' compensation case managers employed by healthcare reimbursement providers often have substantial influence over decisions about post-inpatient services for people with SCI. As such, case managers' relative perceptions about physical therapy services provided via telecommunication technologies and outpatient clinics are of interest. Our purpose for this survey study was to explore the perceptions of workers' compensation case managers regarding the relative advantages and disadvantages of various aspects of physical therapy services provided to people with SCI via telerehabilitation versus clinic-based rehabilitation and the primary factors influencing their service recommendations. Specific research questions included:

1. What is the relation among experiential factors and workers' compensation case managers' likelihood of recommending synchronous telerehabilitation or clinic-based rehabilitation for persons in need of physical therapy services?
2. What aspects of telerehabilitation do workers' compensation case managers identify as being superior or inferior to clinic-based rehabilitation when considering the physical therapy needs of persons with SCI?
3. What is the relative importance of various predetermined factors (i.e., travel convenience for clients, timeliness of treatment initiation, cost savings, clinician expertise, degree of physical disability, and client preference) on workers' compensation case managers' decisions about recommending synchronous telerehabilitation versus clinic-based physical therapy for persons with SCI?

## METHODS

### SURVEY RESPONDENTS

We recruited survey respondents from a database of workers' compensation case managers affiliated with healthcare payors located throughout the US. Each case manager received initial and follow-up emails soliciting participation.

### SURVEY INSTRUMENT

We created the survey instrument using Qualtrics, an online survey platform. The instrument included three sections: demographic information, synchronous telerehabilitation questions, and clinic-based rehabilitation questions. Demographic questions addressed respondents' education, gender, race and ethnicity, age, state of residence, years of work as a case manager, and caseload characteristics regarding size and frequency of working with clients with SCI.

The telerehabilitation and clinic-based rehabilitation sections of the survey included sets of identical questions for each service delivery method. To ensure consistent understanding of service delivery terminology, we defined synchronous telerehabilitation as, “the delivery of rehabilitation services via video-conferencing technology that allows real-time, two-way audiovisual interaction between a client and clinician during treatment sessions,” and clinic-based rehabilitation as, “the delivery of rehabilitation services with the client and clinician in the same physical location.” After defining a service delivery model, initial questions queried a respondent about familiarity with the method and frequency of recommending it for persons with SCI; respondents selected their responses from a 5-point Likert-type rating scale for the familiarity question and a 7-point Likert-type rating scale for the frequency of recommendation question. Next, respondents used 6-point Likert-type scales to respond to 13 statements about service delivery quality and effectiveness, four statements about physical therapist-client interactions and relationships, and four statements about convenience and access to rehabilitation services. Labels for the scale ratings ranged from strongly disagree (i.e., 1) to strongly agree (i.e., 6). The final item within each set required respondents to select from six options the most important and second most important factors influencing their decision to recommend one service delivery model over the other for a client with SCI. The survey then progressed to presentation of the second question set targeting the other service delivery option. The order of question sets was randomized across respondents using survey flow options within Qualtrics.

### DATA COLLECTION AND ANALYSIS

We obtained Institutional Review Board approval prior to recruiting and collecting data from survey respondents. We used e-mail solicitation to distribute invitations to participate in the project.

The Qualtrics platform allowed for compilation of survey responses. As an initial analysis step, we removed any surveys with incomplete responses from the dataset; hence, all analyses only used fully completed surveys. As appropriate, we computed descriptive statistics to characterize respondents' demographic and work experiences. We also separated respondents into three subgroups based on their self-reported familiarity with synchronous telerehabilitation and clinic-based rehabilitation (i.e., low = Likert-type rating of 1 or 2, medium = Likert-type rating of 3, and high = Likert-type rating of 4 or 5). We used these subgroups to determine whether a significant difference existed in respondents' familiarity with the two service delivery options by performing a 3 x 2 chi-square analysis. We determined the effect size of the chi-square result by computing Cramer's V (φ) and using the values of 0.01, 0.03, and 0.05 as minimal criteria for small, medium, and large effect sizes, respectively.

We computed and determined the significance of and effect sizes associated with Spearman rank order correlations between respondents' work experiences and their likelihood of recommending synchronous telerehabilitation or clinic-based rehabilitation for persons in need of physical therapy services. Targeted experiential factors were respondents' (a) years working as a case manager, (b) typical caseload size, (c) typical number of people with SCI served at any given time, (d) familiarity with synchronous telerehabilitation, and (e) familiarity with clinic-based rehabilitation. Our decision to compute Spearman rho correlation coefficients was because our data were ordinal in nature. Coefficients between .10 and .29 corresponded with small effect sizes, those between .30 and .49 corresponded with medium effect sizes, and those greater than .50 corresponded with large effect sizes.

We computed the difference score between each respondent's rating for the likelihood of recommending clinic-based versus synchronous telerehabilitation as a service delivery model. We split these data in accordance with respondents' familiarity ratings with each service delivery model and reported the resulting descriptive statistics. As appropriate, we used the Kruskal-Wallis rank sum test and Dunn-Bonferroni post-hoc analyses to determine the presence of significant differences among familiarity subgroups regarding delivery model recommendation difference scores. Computation of eta-squared allowed effect size evaluation (i.e., small: η2 between 0.01 and 0.059; medium: η2 between 0.06 and 0.139; large: η2 ≥ 0.14).

We used Likert-type scale responses in the next step of our analysis procedures to compute Wilcoxon signed rank tests to determine which aspects of physical therapy case managers identified as significantly superior or inferior based on service delivery model. We used Wilcoxon tests rather than dependent t-tests because computation of Shapiro-Wilk normality tests revealed between-group difference scores that were not distributed normally. Given performance of multiple Wilcoxon tests, we applied the Bonferroni correction to the probability level used to determine significance; the correction yielded a critical p-value of .0024.

Finally, we tallied the number of respondents who selected each of six predetermined factors as most important or second-most important when deciding to recommend synchronous telerehabilitation rather than clinic-based rehabilitation, or vice versa, as a service delivery option. We used the combined most important and second-most important tallies to compute a chi-square analysis to determine the presence of a significant difference; computation of Cramer's V (φ) yielded a value for assessing the effect size magnitude (i.e., small = 0.10, medium = 0.30, large = 0.50). For follow-up analyses, we used Fisher's exact test and an adjusted probability of .01.

## RESULTS

### RESPONDENTS

A total of 118 case managers initiated survey completion, but only 89 of the submitted surveys were completed in full; hence, analyses were based on 89 respondents. As is typical of the population of case managers, all but one respondent identified as female. Eighty-six (96.63%) were White, with the three remaining respondents opting not to report race information; 85 (95.51%) identified as non-Hispanic, two identified as Hispanic, and two opted not to report their ethnicity. Additional demographic information appearing in Table 1 shows that survey respondents ranged in age from 29 to 75 years *(M* = 52.52 years, *Median* = 57 years, *SD* = 14.21), and over half (i.e., 51 of 89) had worked as a case manager for more than 21 years. Almost two-thirds (i.e., 57 of 89) had three or fewer people with SCI on their caseload at any given time, thus attesting to the relative rarity of the condition with regard to worker's compensation claims. Respondents came from 28 states across the US.

**Table 1:** Respondents' Demographic and Work Experience Information

Variable	Number (Percent)
Age in	
≤ 29 years	1 (1.12%)
30 – 39 years	9 (10.11%)
40 – 49 years	13 (14.61%)
50 – 59 years	31 (34.83%)
60 – 69 years	33 (37.08%)
≥ 70 years	2 (2.25%)
Time employed as a case manager	
≤ 5 years	13 (14.61%)
6 – 10 years	14 (15.73%)
11 – 15 years	11 (12.36%)
16 – 20 years	13 (14.61%)
≥ 21 years	38 (42.70%)
Size of caseload	
≤ 10 people	7 (7.87%)
11 – 20 people	17 (19.10%)
21 – 30 people	27 (30.34%)
31 – 40 people	14 (15.73%)
≥ 41 people	24 (26.97%)
Typical number of SCI cases	
0 – 3	57 (64.04%)
4 – 6	17 (19.10%)
7 – 10	10 (11.24%)
≥ 11	5 (5.62%)
Familiarity with	
Synchronous telerehabilitation	
Low	31 (36.67%)
Medium	34 (36.67%)
High	24 (26.67%)
Clinic-based rehabilitation	
Low	4 (5.00%)
Medium	7 (8.33%)
High	78 (86.67%)

Separating respondents into three groups based on their self-reported familiarity with synchronous telerehabilitation and with clinic-based rehabilitation revealed a discrepancy between the two service delivery options. Specifically, approximately equal numbers of respondents indicated low, medium, and high familiarity with synchronous telerehabilitation, but the majority affirmed high familiarity with clinic-based rehabilitation. Computation of a 3 × 2 chi-square analysis confirmed a significant difference in service delivery familiarity, *χ*^*2*^*(2, N* = 89) = 67.20, *p* < .00001. The effect size was large as indicated by a Cramer's *V* value of 0.614.

### RELATIONS AMONG EXPERIENTIAL FACTORS AND SERVICE DELIVERY RECOMMENDATIONS

Table 2 shows Spearman rho correlation results among factors associated with case managers' work experiences and their likelihood of recommending synchronous telerehabilitation or clinic-based rehabilitation. A significant positive correlation with a large effect size emerged between familiarity with synchronous telerehabilitation and the likelihood of recommending that service delivery model for people in need of physical therapy following SCI. The corresponding correlation between familiarity with and recommendation of clinic-based rehabilitation was also significant and had a medium effect size. Other significant positive correlations of note were one with a medium effect size between familiarity with telerehabilitation and clinic-based rehabilitation service delivery models and two with small effect sizes between the number of years a respondent had worked as a case manager and familiarity with synchronous telerehabilitation and between familiarity with clinic-based rehabilitation and the likelihood of recommending telerehabilitation service delivery.

**Table 2:** Spearman Correlation Coefficients and Significance for Case Managers' Work Experience Factors and Likelihood of Recommending Service Delivery Options

	Caseload total size	Caseload SCI size	ST familiarity	CB familiarity	Recommend ST	Recommend CR
Years worked	0.12	−0.02	0.25*	0.19	0.19	−0.04
Caseload total size		0.29**	0.15	0.14	0.11	0.08
Caseload SCI size			.17	.13	0.14	0.18
ST familiarity				.36***	.67****	.09
CB familiarity					.22*	.44****
Recommend ST						0.04

*Note.* ST = Synchronous telerehabilitation services; CB = clinic-based rehabilitation services.

* = *p* < .05;

** = *p* < .01;

*** = *p* < .001;

**** p < .0001

We derived a new variable—the difference score between ratings assigned for the likelihood of recommending clinic-based and synchronous telerehabilitation services—to explore further the significant correlations between service option familiarity and recommendation. Descriptive statistics for the difference scores split by familiarity subgroup appear in Table 3. Because all but a few respondents indicated high familiarity with clinic-based rehabilitation services, we only report descriptive statistics for subgroupings for this type of service delivery; however, the relatively even split among familiarity subgroupings for synchronous telerehabilitation service delivery allowed additional statistical analysis of these data.

**Table 3:** Descriptive Statistics for Familiarity Subgroups Regarding Service Delivery Recommendation Difference Scores

Difference score statistics	Familiarity with synchronous telerehabilitation			Familiarity with clinic-based rehabilitation		
	Low (n = 31)	Medium (n = 34)	High (n = 24)	Low (n = 4)	Medium (n = 7)	High (n = 78)
Mean	3.52	3.18	1.96	0.25	3.00	3.10
Median	4.00	3.50	2.00	0.00	3.00	3.00
Standard deviation	1.67	1.51	1.65	0.50	2.31	1.56

Shapiro-Wilk test computation revealed violation of the normality assumption among the telerehabilitation familiarity subgroups (*W* = 0.929, *p* = .0001), so we computed the nonparametric Kruskal-Wallis rank sum test instead of a parametric statistic. A significant difference with a medium effect size (eta-squared *(η^2^)* between .060 and .139) appeared among the three telerehabilitation familiarity subgroups regarding the difference in likelihood of recommending synchronous telerehabilitation and clinic-based rehabilitation, *χ*^2^(2, *N* = 89) = 11.96, *p* = 0.0025, *η^2^* = 0.116. Subsequent performance of the Dunn-Bonferroni test revealed significant post-hoc analysis differences between the high and low subgroups, *z* = -3.35, *p* = .0024, and the high and medium subgroups, *z* = -2.628, *p* = .0258; the result was not significant between the low and medium subgroups, *z* = 0.846, *p* = 1.00. In both cases with significant results, greater familiarity yielded less difference between the likelihood of recommending telerehabilitation and clinic-based rehabilitation for service delivery to people needing physical therapy. Still, case managers had a greater likelihood of recommending clinic-based rather than telerehabilitation services even when telerehabilitation familiarity was high.

### PERCEPTIONS ABOUT SYNCHRONOUS TELEREHABILITATION AND CLINIC-BASED SERVICES

#### SERVICE DELIVERY QUALITY AND EFFECTIVENESS

**Figure 1:**
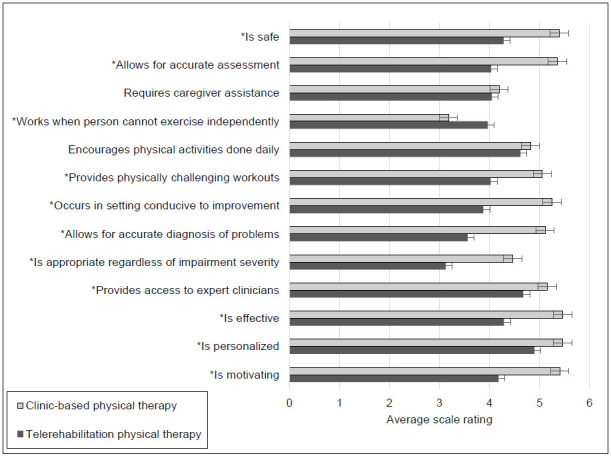
Average Likert-type Scale Ratings Concerning Service Delivery Quality and Effectiveness

*Note.* Error bars represent the standard error of mean.

Higher Likert-type scale ratings indicated more positive perceptions about a treatment approach than lower scale ratings for survey items addressing service delivery quality and effectiveness. As shown in Figure 1, case managers assigned higher ratings to all but one item when considering clinic-based physical therapy services rather than synchronous telerehabilitation physical therapy services. Computation of Wilcoxon tests revealed significant differences for 11 of the 13 items given a probability of less than .0024; test statistics and probability levels for individual survey items appear in the Appendix.

* indicates significant difference between the two service delivery models given *p* < .0024.

#### PHYSICAL THERAPIST-CLIENT INTERACTIONS AND RELATIONSHIPS

**Figure 2:**
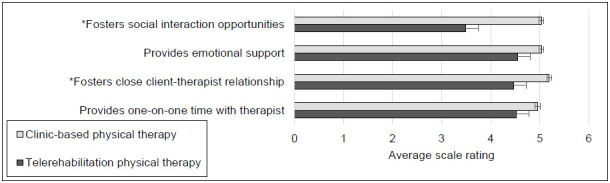
Average Likert-type Scale Ratings Concerning Therapist-Client Interactions and Relationships

*Note.* Error bars represent the standard error of mean.

Four survey items addressed interactions and relationships between a therapist and client. Higher ratings indicated more positive perceptions than lower ratings. As shown in Figure 2, case managers consistently assigned higher ratings when considering clinic-based delivery rather than synchronous telerehabilitation delivery of physical therapy services. Computation of Wilcoxon tests revealed significant differences for 2 of the 4 items given a probability of less than .0024; test statistics and probability levels appear in the Appendix.

* indicates significant difference between the two service delivery models given *p* < .0024.

#### CONVENIENCE AND ACCESS TO REHABILITATION SERVICES

**Figure 3:**
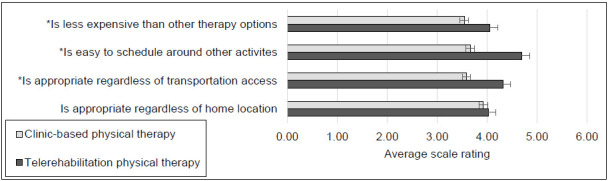
Average Likert-type Scale Ratings Concerning Convenience and Access to Physical Therapy Rehabilitation Services

*Note.* Error bars represent the standard error of mean.

Four survey items addressed convenience and access to physical therapy rehabilitation services. Once again, higher ratings indicated more positive perceptions than lower ratings. Figure 3 provides a graphic display of the average Likert-type scale ratings assigned for clinic-based and telerehabilitation service delivery. In contrast to other survey sections, computation of Wilcoxon tests revealed that case managers assigned significantly higher ratings for synchronous telerehabilitation service delivery than clinic-based service delivery for 3 of the 4 items given a probability of less than .0024; test statistics and probability levels appear in the Appendix.

* indicates significant difference between the two service delivery models given *p* < .0024.

### FACTORS AFFECTING DECISION-MAKING ABOUT SERVICE DELIVERY RECOMMENDATIONS

Table 4 shows tallies of respondents' selections of most important and second-most important factors influencing their choice to recommend one service delivery option over the other. By combining the most and second-most important tallies, the degree of a client's physical disability and clinician expertise emerged as the most frequently cited factors for recommending clinic-based rehabilitation over synchronous telerehabilitation. In contrast, travel convenience was the most frequently cited factor for recommending synchronous telerehabilitation over clinic-based rehabilitation, although physical disability and clinician expertise were also common. Cost savings was the least frequently cited factor for recommending either service delivery model. Chi-square computation revealed a significant difference with a small effect size among the factors selected for the two service delivery options, *χ*^2^(5, *N* = 178) = 13.204, *p* = .0215, *φ_c_* = 0.193. Post-hoc analysis with Fisher's exact test and a probability level of .01 revealed a significant result only between travel convenience and the other factors, *p* = .0007.

**Table 4:** Number of Respondents Identifying Various Factors as Most and Second Most Important when Recommending Synchronous Telerehabilitation versus Clinic-based Rehabilitation

	Synchronous telerehabilitation			Clinic-based rehabilitation		
	Most important	2^nd^ most important	Sum	Most important	2^nd^ most important	Sum
Degree of physical disability	23	24	47	28	24	52
Clinician expertise	26	14	40	33	19	53
Travel convenience	26	25	51	6	18	24
Client preference	6	14	20	13	12	25
Speed of treatment initiation	8	7	15	8	13	21
Cost savings	0	5	5	1	3	4

## DISCUSSION

Synchronous telerehabilitation remains unfamiliar to a majority of case managers despite its increased use as a service delivery option because of physical contact restrictions imposed in response to the COVID-19 pandemic. Only about one in four case managers affiliated with healthcare payors for workers' compensation claims reported being very familiar or extremely familiar with synchronous telerehabilitation, whereas about eight of every nine case managers affirmed that level of familiarity with clinic-based rehabilitation. Thus, despite decreased availability of clinic-based physical therapy services forced by COVID-19 restrictions and promotion of telerehabilitation as a viable alternative service delivery model (Lee, 2020; Stein et al., 2020; Turolla et al., 2020), most case managers remain unfamiliar with the latter option.

Case managers' lack of familiarity with synchronous telerehabilitation means that both additional research documenting advantages and disadvantages and additional educational opportunities to inform professionals about benefits and detriments associated with various service delivery models are warranted. Extant research and professional training appear insufficient to ensure case managers and other professionals recognize that people derive comparable physical therapy benefits for many aspects of clinic-based and synchronous telerehabilitation services. This was evident in the disparate ratings survey respondents assigned to the two models, particularly with regard to service quality, effectiveness, convenience, and access. In fact, with the exception of being easy to schedule around other activities and minimizing transportation barriers, the obtained ratings opposed research findings documenting a telerehabilitation advantage for providing access to expert clinicians (Irgens et al., 2018; Niknamian, 2019; Schmeler et al., 2008), being appropriate regardless of home location (Dunphy & Gardner, 2020), and occurring in a comfortable and private setting conducive to physical progress (Kairy et al., 2013; Theodoros & Russell, 2008). For other issues—such as being safe, yielding accurate assessments and diagnoses, being effective, providing personalized treatment, and fostering client motivation—existing research supports similarity between the two delivery models (Cramer et al., 2019; Kairy et al., 2013; Levy et al., 2015; Neo et al., 2019; Theodoros & Russell, 2008; Turolla et al., 2020) that was not realized in the current respondents' ratings.

Correlation findings from the obtained dataset further support the importance of educating case managers about service delivery options for people likely to benefit from outpatient physical therapy. Specifically, respondents reporting familiarity with synchronous telerehabilitation recommended it to their clients more frequently than those without such familiarity. Telerehabilitation familiarity corresponded with a greater number of years working in the profession as well as greater familiarity with clinic-based rehabilitation. Collectively, these findings confirm the need for continued research evaluating the value and appropriateness of telerehabilitation as a service delivery option. Assuming positive findings, additional educational activities should follow to enhance professionals' knowledge about and acceptance of telerehabilitation as a worthwhile and appropriate service delivery option.

Several ways of attaining familiarity with synchronous telerehabilitation as a service delivery option are possible. Although we asked survey respondents about their familiarity with synchronous telerehabilitation, we did not ask them where or how they gained that familiarity. Hence, we do not know the preferred or most common methods of gaining knowledge about service delivery options. Possibilities include attending conferences or workshops, pursuing independent professional reading, or conversing with colleagues about available resources. Because some methods of disseminating such information may be more effective than others, future researchers may wish to investigate the relative value of each.

## LIMITATIONS

A limitation of the study reported herein was that we only surveyed case managers who worked for healthcare payors dealing with workers' compensation claims. This represents an important subgroup of case managers for people with SCI because many such cases result from injuries sustained while performing job duties. However, many other cases of SCI result from accidents not associated with work settings and not resulting in workers' compensation claims. Determining the familiarity and perceptions of case managers not dealing with workers' compensation claims may reveal different results from those expressed by the current study respondents.

Another study limitation was that we did not ask survey respondents to explain any differences they reported in their perceptions about synchronous telerehabilitation and clinic-based rehabilitation service delivery models. Such explanations may provide important clues about factors contributing to misperceptions about telerehabilitation practices or reasons why case managers are reluctant to recommend telerehabilitation physical therapy as often as clinic-based treatment. Exploring these issues will be critical to advancing acceptance of synchronous telerehabilitation.

## CONCLUSION

Telerehabilitation as a service delivery model originally stemmed from a need to provide services to people in remote locations that prevented easy access to brick-and-mortar facilities. Over subsequent years, however, the value of synchronous telerehabilitation became increasingly apparent. Not only is using technology to access services remotely essential in some circumstances, but it is also a viable and equal alternative to clinic-based service delivery in many respects (Cramer et al., 2019; Dunphy & Gardner, 2020; Irgens et al., 2018; Kairy et al., 2013; Levy et al., 2015; Neo et al., 2019; Niknamian, 2019; Schmeler et al., 2008; Theodoros & Russell, 2008; Turolla et al., 2020). However, knowledge about synchronous telerehabilitation among case managers responsible for service coordination is less widespread than that about clinic-based services. The result is persistence of the belief that clinic-based service delivery is a superior option for most people in need of physical therapy interventions. Additional research and education about the efficacy and feasibility of synchronous telerehabilitation is the next step in establishing its acceptance as a service delivery model.

## APPENDIX WILCOXON VALUES AND PROBABILITY RESULTS FOR SURVEY ITEMS

**Table:**

Survey item	Mean		Test statistic	*p*-value
	CB	ST		
Is safe	5.39	4.28	98.0	< .0001*
Allows for accurate assessment	5.36	4.03	72.0	< .0001*
Requires caregiver assistance	4.19	4.04	587.5	.3537
Works when person cannot exercise independently	3.18	3.97	1019.5	< .0001*
Encourages physical activities done on daily basis	4.82	4.62	548.0	.1228
Provides physically challenging workouts	5.06	4.02	84.0	< .0001*
Occurs in setting conducive to improvement	5.25	3.88	100.0	< .0001*
Allows for accurate diagnosis of problems	5.11	3.56	160.0	< .0001*
Is appropriate regardless of impairment severity	4.46	3.12	198.0	< .0001*
Provides access to expert clinicians	5.16	4.67	420.0	.0008*
Is effective	5.46	4.29	93.0	< .0001*
Is personalized	5.46	4.90	336.0	< .0001*
Is motivating	5.40	4.18	75.0	< .0001*
Fosters social interaction opportunities	5.03	3.48	120.0	< .0001*
Provides emotional support	5.03	4.55	413.5	.0032
Fosters close client-therapist relationship	5.19	4.47	268.0	< .0001*
Provides one-on-one time with therapist	4.96	4.52	476.0	.0037
Is less expensive than other therapy options	3.54	4.06	879.0	.0006*
Is easy to schedule around other activities	3.66	4.79	1548.5	< .0001*
Is appropriate regardless of transportation access	3.58	4.31	1394.0	.0008*
Is appropriate regardless of home location	3.92	4.02	813.5	.6421

*Note*. Significant differences between the two service delivery models required *p* < .0024. CB = clinic-based telerehabilitation; ST = synchronous telerehabilitation.
